# Early preoperative progressive pneumoperitoneum for a symptomatic giant abdominal incisional hernia

**DOI:** 10.1016/j.ijscr.2022.107028

**Published:** 2022-04-02

**Authors:** Camilo A. Polanía-Sandoval, Alejandro Velandia-Sánchez, Carlos J. Pérez-Rivera, Juan Pablo Garcia-Mendez, Felipe Casas-Jaramillo, Paulo A. Cabrera-Rivera

**Affiliations:** a General Surgery Research Group, Fundación Cardioinfantil-Instituto de Cardiología, Bogotá, Colombia; b Universidad del Rosario, School of Medicine and Health Sciences, Bogotá, Colombia

**Keywords:** PPP, Preoperative Progressive Pneumoperitoneum, CeDAR, The Carolinas Equation for Determining Associated Risk, Case report, Abdominal hernia, Preoperative procedure, Pneumoperitoneum

## Abstract

**Introduction and importance:**

Early preoperative progressive pneumoperitoneum (PPP) is a technique that helps large eventrations with loss of domain to reintroduce protruded organs. However, a standardized technique has not been developed. This technique has been proved in elective patients, but the evidence is scarce in patients with a high risk of incarceration/strangulation.

**Clinical findings and investigations:**

We present a 61-year-old patient with history of a thoracoabdominal aneurysm repair, developed a massive incisional hernia with loss of domain. At admission, he presented with abdominal pain and inability to reduce the hernia by himself, however it reduced after clinical examination. Aortic syndromes were excluded.

**Interventions and outcome:**

After a multidisciplinary meeting, early PPP was initially performed. Later he was taken to surgery and admitted in the ICU to prevent abdominal hypertension. Medical complications resolved within 14 days. The patient did not report long-term complications.

**Relevance and impact:**

PPP is a technique that pursues the prevention of abdominal hypertension syndrome in patients with large hernias with loss of domain electively. For patients with high risk of hernia complications, the evidence is limited regarding the applicability of early PPP. A multidisciplinary team can improve decision making and therefore reduce the risk of long-term complications. We show a case where PPP was performed in an acute painful, reducible hernia with a high risk of incarceration, showing that this approach can be an option for acutely ill patients.

## Highlights

• Early PPP in acutely herniated patients can be feasible for non-elective surgery.
• Early PPP can be used to prevent complications like abdominal hypertension.
• A multidisciplinary approach and experts committee are useful for decision making.

## 1 Introduction

Incisional hernias are among the most frequent complications of abdominal surgery [1]. When this condition becomes chronic, it is associated with significant volumetric growth, leading to abdominal hypertension and loss of domain, ensuring prompt surgical intervention. Surgical repairment of chronic abdominal wall eventrations with loss of domain represents a challenge for the surgeon. Reintroducing the extruded contents into the abdominal cavity may cause catastrophic complications such as abdominal compartment syndrome, precipitating multi-organ failure, including acute respiratory failure [2].

Preoperative progressive pneumoperitoneum (PPP) is a strategy that has been proposed to prevent the development of potentially fatal complications. Goñi Moreno described it in 1940, and its primary function is to promote a volumetric expansion of the abdominal cavity, leading to a physiological reintroduction of the abdominal organs and a less challenging and more tolerable surgical procedure [3]. Current scientific consensuses suggest that PPP should be used as adjuvant therapy for elective procedures associated with lower surgical complications and morbidity [4], [5]. However, evidence supporting this technique in patients with acute symptoms requiring stabilization and early intervention in the same hospitalization is scarce. Therefore, we present a case report about the advantages of early PPP in symptomatic giant eventration in a fourth-level health care center. This case report has been reported in line with the SCARE Criteria [6].

## 2 Case presentation

**Fig. 1:**
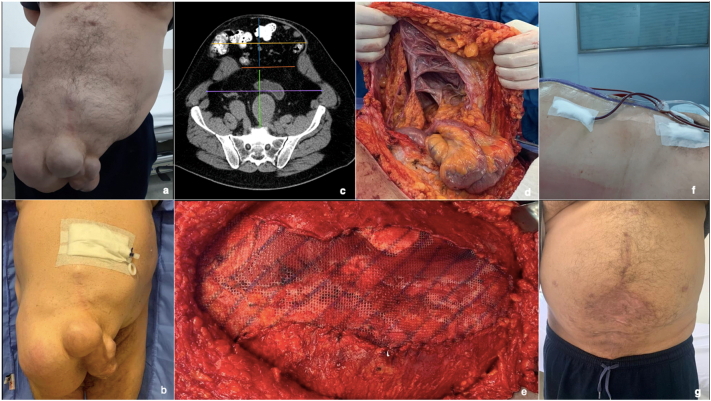
Trans-operative record of the abdominal wall reconstruction. a) Clinical presentation of the patient showing the abdominal incisional hernia. b) Abdominal wall incisional hernia before surgery once the maximum PPP insufflation level was reached. c) Preoperative Axial Computed Tomography. d) Intraoperative finding consisting of adequate intestinal perfusion, with no signs of necrosis or suffering. The adhesions and the multiple sub-eventrations are also visible. e) Polypropylene mesh suitably positioned. f) immediate postoperative result after abdominal wall reconstruction. g) 2-year outcome of abdominal wall reconstruction.

A 61-year-old Caucasian male patient presented to the emergency room with sudden onset of severe diffuse abdominal pain associated with a presyncope episode, hypertension, dehydration, and tachycardia. Past medical history revealed obesity (BMI of 31,5 kg/m^2^), hypertension, and a thoracoabdominal aortic aneurysm repair 9-years prior to the current consultation. Physical examination showed a massive abdominal incisional hernia with multiple sub-eventrations (Fig. 1a), indurated and painful on palpation, with a slight purple coloration. On physical examination the content of the hernia was successfully reduced. An emergent Computed Angio-tomography showed an abdominal aortic aneurysm with a non-complicated chronic dissection extended to iliac arteries, ruling out an acute vascular condition. Laboratory tests showed no further abnormalities.

The case was immediately discussed at the institutional abdominal wall committee due to the patient's acute condition, multiple comorbidities, imminent risk of strangulation, and an 81% predicted risk of complications by The Carolinas Equation for Determining Associated Risk (CeDAR). Only CeDAR-Score was obtained due to the unnecessity of emergent primary repair but the high risk of out-patient complications if an elective repair was considered (Table 1).

**Table 1:** Committee reasoning for decision making of the best approach for the patient.

Committee reasoning for PPP and hernia repair in the same hospitalization
The patient was stable as complete reduction was achieved without signs of incarceration or loop distress.
High risk of out-patient incarceration or strangulation.
The possibility of correction with PPP in a patient with a history of an aneurysm with immediate vascular surgery consult in case of needing it.
Vascular surgery ruled out acute aortic pathology
Availability of interventional radiology to do PPP catheterization guided through echography.
In-hospital follow-up and close monitoring of PPP according to tolerance to pneumoperitoneum.
Abandonment of PPP in case of acute exacerbation of the hernia.
Surgery in conjunction with plastic surgery for defect correction
ICU for risk of abdominal hypertension

It was decided that the patient would benefit of PPP because resolution of acute symptoms, high risk of hernia complication, and history of aortic pathology, requiring in-hospital surveillance (Table 2). After PPP, subsequent elective surgical repair in-hospital (no emergency surgery) was planned. Surgical planning was intended to ensure a better functional and quality of life results. The surgery was leaded and performed by a general surgeon with special training in abdominal wall repair. If the patient presented new acute onset hernia symptoms like pain or signs of complication, emergent surgery should be performed.

**Table 2:** Risks and benefits of performing PPP in acutely ill patients with giant abdominal incisional hernias.

Benefits of PPP	Potential risks of PPP
Availability of a multi-disciplinary approach by abdominal wall group led by an expert surgeon	Acute complications of PPP
Stay in an fourth level hospital with core in vascular care	Patient obesity
Clinical team support due to complex surgical background	Previous medical history of thoracoabdominal anuerysm repair
Integrated surgical intervention with the abdominal wall team, interventional radiology, plastic and vascular surgery	Risks associated to polonged hospitalization
Close monitoring and surveillance for PPP insufflation response.	
*All of this considerations were evaluated in the abdominal wall committee by means of a multidisciplinary approach	

The patient underwent intraperitoneal catheter placement by interventional radiology, and PPP was initiated, with a maximum insufflation of 10,000 ccs (Fig. 1b). Air insufflation was progressively increased based on patient tolerance, with an average of 833 ccs per day. There were no clinical or laboratory signs of deterioration. According to literature and patient tolerability to pneumoperitoneum, surgery was performed on day 12 after the total volume was achieved.

Under general anesthesia, an incision was made around the laparotomy scar. A 10 cm composite local skin flap stalk was started in all directions until the healthy fascia was identified and the herniated sac was opened. Adequate integrity of the small and large bowels was assured (Fig. 1d). All adhesions were released, and a prophylactic appendectomy was performed.

Subsequently, the Ramirez technique with the Carbonell maneuver was used to reconstruct the abdominal wall. The entire sac was resected, leaving the medullary muscle complexes of the rectus abdominis muscle borders free. The local cutaneous flap stalk of the aponeurotic muscle was continued, freeing the crescent line on both sides, and identifying and separating the more significant oblique muscle from the lesser oblique bilaterally. This stem of flaps allowed closure at the midline, achieving a release of 8 cm on both sides; this was determinant for the primary closure of the midline.

The primary midline closure was performed with PDS 0 stitches “in X” technique. The midline was reinforced with a continuous suture with the same material (PDS 0 stitches), starting at each GAP angle and knotted in the middle third. A 40 × 40 cm medium density polypropylene mesh was placed between the oblique muscles at each angle with cardinal fixations with PDS 0 stitches, and the reconstruction of the semilunar newline was performed (Fig. 1e). Fixing the oblique muscle to the polypropylene mesh was done to ensure the correct functioning of all the restored muscles of the abdominal wall. Subsequently, the procedure was continued by plastic surgery, which closed the remaining tissues and conducted several flaps for the excess skin (Fig. 1f).

The patient was treated in an intensive care unit in the immediate postoperative period. He presented postoperative dyspnea and desaturation with Venturi at 50%. A bedside chest radiography showed lower left atelectasis and bilateral pleural effusions. Orotracheal intubation was decided to prevent respiratory depression and muscular fatigue. Simultaneously, the patient presented a multifactorial acute kidney injury classified as a KDIGO III. Renal replacement therapy was initiated. Acute abdominal hypertension was suspected. After several days of aggressive medical management, the patient presented a good evolution, and mechanical ventilation and hemodynamic support were successfully weaned.

The patient showed satisfactory in-hospital evolution and was discharged 14 days after surgery. After a two-year follow-up, the patient presented no complications, and an adequate esthetic evolution was observed (Fig. 1g). Also, the patient referred an improvement in his quality of life in his postoperative controls with good tolerability of the procedure.

## 3 Discussion

Loss of domain refers to the apparent impossibility of reintroducing the organs of the hernia sac into the abdominal cavity. This phenomenon is caused by two simultaneous processes: the reduction of volume in the abdominal cavity and the increase of volume in the hernia sac. Based on computed tomography, radiological methods have been proposed to determine the gap between the volume of the hernia contents and that of the abdominal cavity, thus elucidating how much the volume of the latter should be increased before surgical repair. The ratio between these two parameters (hernia sac volume/abdominal cavity volume) was described in 2010, known as the Tanaka index [7].

Recent publications have shown the experience with PPP in cases of hernias with loss of domain [8]. However, the patients included in these studies were clinically stable and received elective procedures [7], [8], [9]. Additionally, there is currently no consensus about the amount of gas introduced into the abdominal cavity [9]. Therefore, literature on the preparation and repair in patients with giant abdominal incisional hernia with loss of domain and acute clinical symptoms is insufficient. Careful selection of the patient and individualized approach is mandatory due to the variety of presentations.

In this case report, the risk was greater to program the surgery than the benefit of out-hospital environment (Table 2), and careful in-hospital surveillance was the best option. Therefore, we considered PPP due to initial stabilization of acute process (no complications after manual reduction), its non-elective but not emergent need to treat the condition, and past medical history of aortic pathology. The abdominal wall committee decision was based on the aspects resumed in Table 1. The volume of pneumoperitoneum was controlled in terms of the best actual evidence. Complications of pneumoperitoneum were absent, and abdominal hypertension syndrome was present in the post-operatory, but at a fewer level, making it more plausible to treat.

In our experience, PPP can be a valuable tool in patients requiring immediate wall reconstruction. It is always necessary to perform adequate and individualized surgical planning for each patient and rely on an interdisciplinary team and a well-established abdominal wall group to minimize the possibility of adverse events [10]. This case shows that this technique can reduce the side effects of reintroducing abdominal content of these types of hernias; however, different approaches can be made because of an unstandardized way to do it. The patient showed signs of abdominal hypertension; however, it was limited and successfully treated, leading to no long-term complications, maybe because of the use of PPP. More studies are needed to assess the probability of reducing this complication using PPP, but PPP can be a preventive measure in patients with large hernias with loss of domain [11].

## 4 Conclusion

Further and more robust analytical studies are required. Other adjuvant tools different from PPP should be evaluated in managing patients that require an early intervention of giant abdominal incisional hernias.

## Provenance and peer review

Not commissioned, externally peer-reviewed.

## Funding

This research did not receive any specific grant from funding agencies in the public, commercial, or not-for-profit sectors.

## Consent for publication

Written informed consent for publication was obtained from the patient for this case report.

## Research registration

None.

## Guarantor

Paulo A. Cabrera MD, MSc.

## CRediT authorship contribution statement

CAP: Conceptualization, Investigation, Writing- Original Draft and Visualization AV: Conceptualization, Investigation and Writing- Original Draft CJP: Supervision, Writing – Reviewing and Editing, Validation JPG: Conceptualization, Investigation and Writing- Original Draft FC: Supervision, Writing – Reviewing and Editing PAC: Supervision, Writing – Reviewing and Editing, Validation and Project Administration.

## Declaration of competing interest

The authors certify that they have no affiliations with or involvement in any organization or entity with any financial interest in the subject matter or materials discussed in this manuscript.
